# A performance comparison of static VAr compensator based on Goertzel and FFT algorithm and experimental validation

**DOI:** 10.1186/s40064-016-2034-7

**Published:** 2016-03-31

**Authors:** O. Fatih Kececioglu, Ahmet Gani, Mustafa Sekkeli

**Affiliations:** Department of Electrical and Electronics, Faculty of Engineering, Kahramanmaras Sutcu Imam University, Kahramanmaras, Turkey

**Keywords:** Static VAr compensators, FFT, Goertzel algorithm, Reactive power control

## Abstract

The main objective of the present paper is to introduce a new approach for measuring and calculation of fundamental power components in the case of various distorted waveforms including those containing harmonics. The parameters of active, reactive, apparent power and power factor, are measured and calculated by using Goertzel algorithm instead of fast Fourier transformation which is commonly used. The main advantage of utilizing Goertzel algorithm is to minimize computational load and trigonometric equations. The parameters measured in the new technique are applied to a fixed capacitor–thyristor controlled reactor based static VAr compensation system to achieve accurate power factor correction for the first time. This study is implemented both simulation and experimentally.

## Background

Reactive power compensation is a very important issue in the expansion planning and operation of electrical power systems. Traditional solution of the compensation is to use fixed-capacitors or reactors for providing and absorbing reactive power respectively (Miller 1982; De La Rosa 2006). Another way of the compensation is to use static VAr compensator (SVC) to compensate reactive power. Since SVC system is fast, smooth and reliable, it has been widely used in power systems (Gelen and Yalcinoz 2010; Mathur and Varma 2002; Lee et al. 2001; Uzunoglu and Onar 2008).

Static VAr systems are generally used in the fast changing events such as arc furnaces and steel industry where the current alters rapidly (El-Saady 2001). This type of industry and rising population of power electronics-based loads causes the increase of harmonic distortion in the power systems. A large proportion of the industrial, commercial and domestic loads are now non-linear. The more use of nonlinear loads has increased harmonic pollution on the power system (De La Rosa 2006). This causes to complicate measuring and calculating the fundamental components of active and reactive power. It is needed to be developed more precise measurement method for existing distortions.

The main problem is how these power components are properly described in non-sinusoidal conditions. In the case of non-sinusoidal current and voltage, it becomes essential to use signal processing methods for power measurement. For this purposes, Fourier transform-based methods are usually used for analyzing power system harmonics. Fast Fourier transformation (FFT) algorithm is generally used for these purposes (Ozdemir and Ferikoglu 2004; Sekkeli 2005). In this study, Goertzel algorithm is preferred to utilize according to FFT algorithm to measure fundamental components of the active and reactive power. Goertzel algorithm is superior with respect to FFT algorithm to include less trigonometric equations (Sekkeli and Tarkan 2013).

In this study, power components measured and calculated by using Goertzel algorithm has been applied in FC–TCR based on SVC system in order to achieve fast and accurate reactive power compensation. SVC system is studied in single-phase under static load conditions. This work is performed both simulation and experimentally. In previous studies, power measurement by using Goertzel algorithm is seriously investigated by some researchers. It is summarized a related study below. Ozdemir and Ferikoglu (2004) worked on a new technique for power measurement. In their study power components are measured by using Goertzel algorithm. Najafi and Yatim (2011) have worked on a static compensator. In that study fundamental components of the power are obtained from the Goertzel algorithm and applied to a static synchronous compensator (STATCOM).

This paper is organized as follows: “Basic FC–TCR based SVC configuration” section briefly presents theory about the TCR–FC based SVC systems. The measurement theory of the Goertzel algorithm is discussed in “The measurement principle” section. “Simulation study and results” section, presents the simulation study and the results obtained for the SVC; in “Experimental results” section includes experimental studies. The main contributions of this paper are summarized in “Conclusions” section.

## Basic FC–TCR based SVC configuration

A SVC is a system capable of rapid compensation. This system can be used for several purposes such as, voltage control, system stability, increasing system capacity and reactive power compensation. SVC can quickly supply variable reactive power to the system using supervising the firing angles of thyristors (Ke et al. 2010). Most of the current thyristor-controlled SVCs are based on providing variable shunt impedance. It can be provided switching shunt capacitors and/or reactors by synchronously in order to control changeable reactive power. Desired reactive power can be achieved by the coordination of switching of capacitor and reactor properly (Kodsi et al. 2006; Liu et al. 2012; Zhijun et al. 2009; Kazemi and Badrzadeh 2004). The main types of SVCs used generally can be identified as follows. Saturated reactor (SR), thyristor controlled reactor (TCR), fixed capacitor–thyristor controlled reactor (FC–TCR), thyristor switched capacitor (TSC) and thyristor controlled reactor–thyristor switched capacitor (TCR–TSC) (Kassem 2012; Petersa et al. 2010).

This paper deals with the FC–TCR based SVC. The working principle of TCR is controlling the firing angle of the thyristor so that adjusting the current in the reactor. TCR current is pure inductive reactive and with 90° lagging. If TCR is combined with a proper capacitor, the compensation system can produce reactive power as required (Kazemi and Badrzadeh 2004; Hooshmand and Esfahani 2011). The circuit mainly consists of two thyristors connected in parallel opposite each other with a series reactor (TCR) and fixed capacitor in parallel (FC). These systems are connected in delta in the 3-phase implementations (Rao et al. 2009). The basic components of the reactor currents through the control of thyristor firing angle is so adjusted that, at the output of the reactive power of the entire system whether lagging or leading (Teleke et al. 2008).

## The measurement principle

The measurement process is very important for SVC system. In order to achieve fast and accurate reactive power compensation, measurement and calculation process has to be performed precisely and accurately (Ozdemir and Ferikoglu 2004). Active, reactive power and power factor parameters of the load and power sources are computed using current and the voltage measured from the system (Novotny and Sedlacek 2009). Because of the non-sinusoidal form of the voltage and current pattern, signal processing methods are very important to calculate the fundamental component of the power (Wang et al. 2008). FFT algorithm is utilized in industrial application (Ozdemir and Ferikoglu 2004).

In this study, in order to calculate fundamental harmonic of the signal, Goertzel algorithm is preferable utilized instead of FFT. In order to find the first harmonic of the signals. It can be shown that Goertzel is preferable algorithm according to FFT in respect to requiring less calculation (Proakis and Manolakis 1996). While Goertzel algorithm is required a few points of the frequency spectrum, FFT is needed whole spectrum of the signals (Chaparro 2010; Shenoi 2006). Fallowing values are obtained when Goertzel algorithm is compared with to a direct N-point discrete Fourier transform (DFT). Goertzel algorithms are required 50 % multiplication, the same number of real additions, and nearly 1/N number of trigonometric equations according to DFT algorithm (Najafi and Yatim 2011). Because the algorithm is performed in the time domain, the application process begins with the arrival of the first signal. On the contrary, DFT must obtain whole spectrum in order to begin the computations. When these two algorithms are compared in respect to a number of multiplication, addition, and trigonometric equations, Goertzel need N multiplication, 2N addition and two trigonometric equations response to DFT, which needs 2N multiplications, 2N addition and 2N trigonometric equations per frequency. The Goertzel algorithm is obtained from usual DFT equation as below (Ozdemir and Ferikoglu 2004).

### Power calculation of the γ th harmonic by the Goertzel algorithm

When a power supply voltage, current and fundamental frequency are donated *v*(*t*), *i*(*t*) and *f*_0_ respectively, general equations are given as follows (Ozdemir and Ferikoglu 2004):

1 $$v(t) = V_{o} + \sum\limits_{\gamma = 1}^{m} {\left[ {a\gamma \cos \left( {\gamma w_{o} t} \right) + b_{\gamma } \sin \left( {\gamma w_{o} t} \right)} \right]}$$

and

2 $$i(t) = I_{o} + \sum\limits_{\mu = 1}^{n} {\left[ {a_{\mu } \cos \left( {\mu w_{o} t} \right) + b_{\mu } \sin \left( {\mu w_{o} t} \right)} \right]}$$

The analog waveforms of the current and voltage should be sampled at discrete-times $t = kT;k \in Z$ and analog signals can be converted to digital signals (Proakis and Manolakis 1996). Then *v*(*t*) and *i*(*t*) are obtained from the continuous-time waveforms *v*(*t*) and *i*(*t*) as,

3 $$v(k) = V_{o} + \sum\limits_{\gamma = 1}^{m} {\left[ {a_{\gamma } \cos \left( {\gamma w_{o} kT} \right) + b_{\gamma } \sin \left( {\gamma w_{o} kT} \right)} \right]}$$

4 $$i(k) = I_{o} + \sum\limits_{\mu = 1}^{n} {} \left[ {\alpha_{\mu } \cos \left( {\mu w_{o} kT} \right) + \beta_{\mu } \sin \left( {\mu w_{o} kT} \right)} \right]$$

For non-sinusoidal signals, Active and reactive power of the *γ*-th harmonics can be produced by the below equations respectively

5 $$P_{\gamma } = 0.5\left( {a_{\gamma } \alpha_{\gamma } + b_{\gamma } \beta_{\gamma } } \right)$$

6 $$Q_{\gamma } = 0.5\left( {a_{\gamma } \beta_{\gamma } - \alpha_{\gamma } b_{\gamma } } \right)$$

In Eqs. (5) and (6), for *γ* = 1, the coefficients *a*_1_, *b*_1_, *β*_1_ and *α*_1_ should be computed in order to find the first component of the active and reactive power from the equations *v*(*k*) and *i*(*k*), by utilizing the Goertzel algorithm (Rao et al. 2009).

## Simulation study and results

In this section, the design of TCR–FC based SVC compensation system is simulated by using the Matlab/Simulink 7.13. Simulation is realized concerning to a single-phase SVC system with a static load model as a parallel R–L load. AC voltage source contains various distorted waveforms including those containing harmonics order of 3, 5, 7, 11. The power system and simulation parameters are listed in Table 1.

**Table 1:** Parameters of simulation and power system

Line frequency	*f*	50 Hz
Line impedance	*L*_*s*_	0.5 mH
	*R*_*s*_	0.1 Ω
Simulation step time	*T*_*s*_	50 µs

**Fig. 1:**
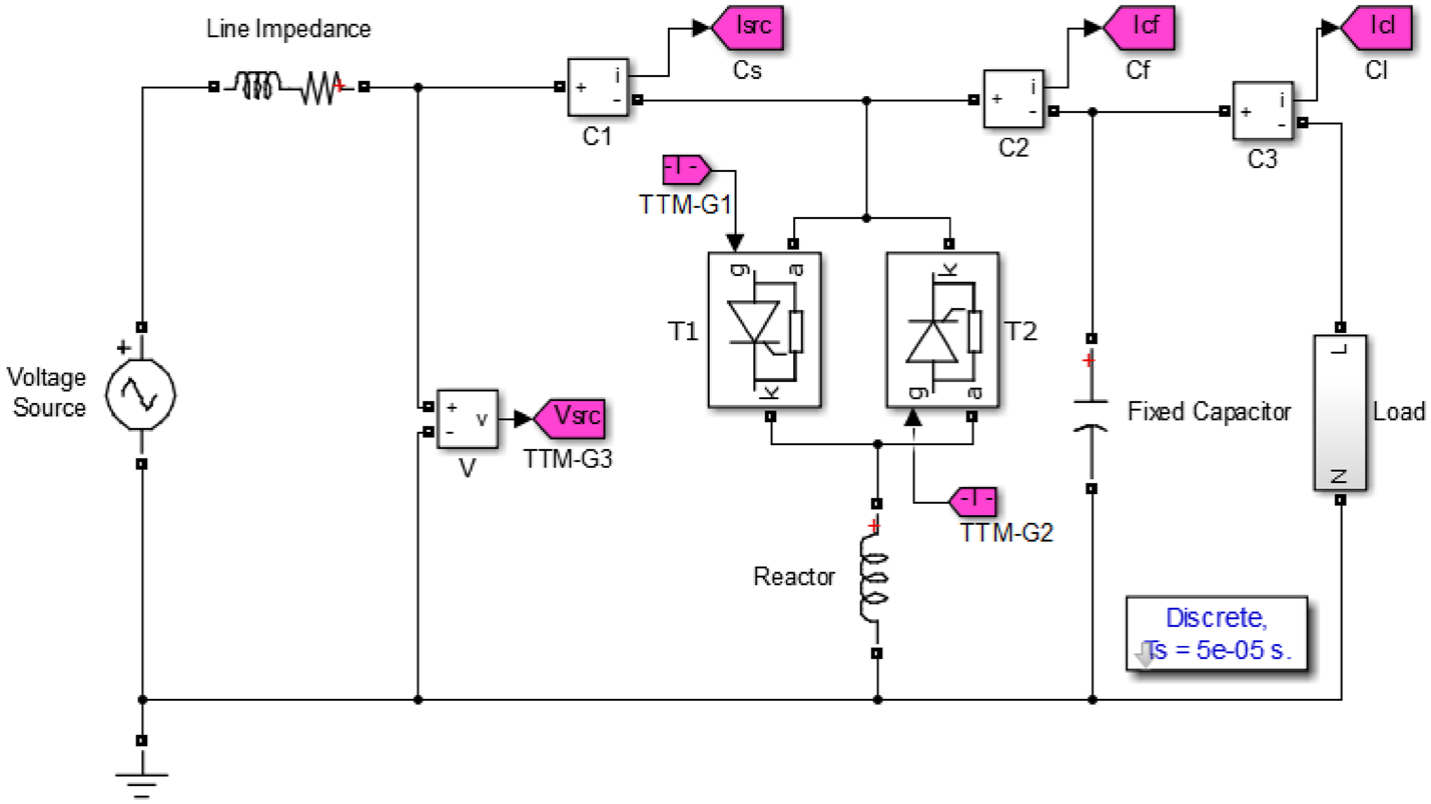
Schematic diagram of fixed capacitor–thyristor controlled reactor (FC–TCR)

Simulation is modeled and performed three different static load groups separately. Load values, SVC settings and results obtained for simulation are given as tables. In order to obtain the effects of the SVC system, simulations are performed separately for the two different states. First it is assumed that there is no SVC in the system. Then, the SVC is put into the system. According to the before and after compensation, fundamental components of voltage and currents are measured and calculated by using both Goertzel and FFT algorithms separately. The structure diagram of simulation study is given Fig. 1.

Fundamental components of the power are calculated by using both Goertzel and FFT algorithms respectively. Computation times were calculated for each algorithm within the sampling period.

**Fig. 2:**
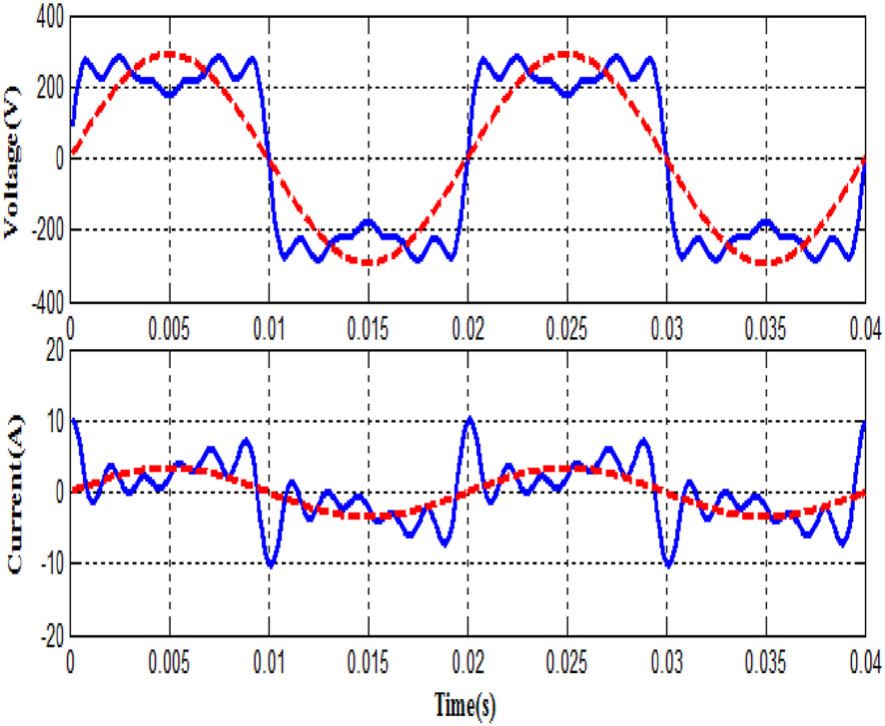
Fundamental component of the voltage and current

It is explicitly illustrated from Fig. 2 that the SVC system is successfully analyzed and obtained a fundamental component of the voltage and current which is normally con-tent harmonics. As it is also clearly seen in this Fig. 5 that the voltage and current waveforms in the load become close to a sinusoidal form after using by Goertzel and FFT algorithm. These fundamental components of the voltage and current are utilized in order to calculate the firing angle of the thyristors for SVC system and active, reactive power components.

**Fig. 3:**
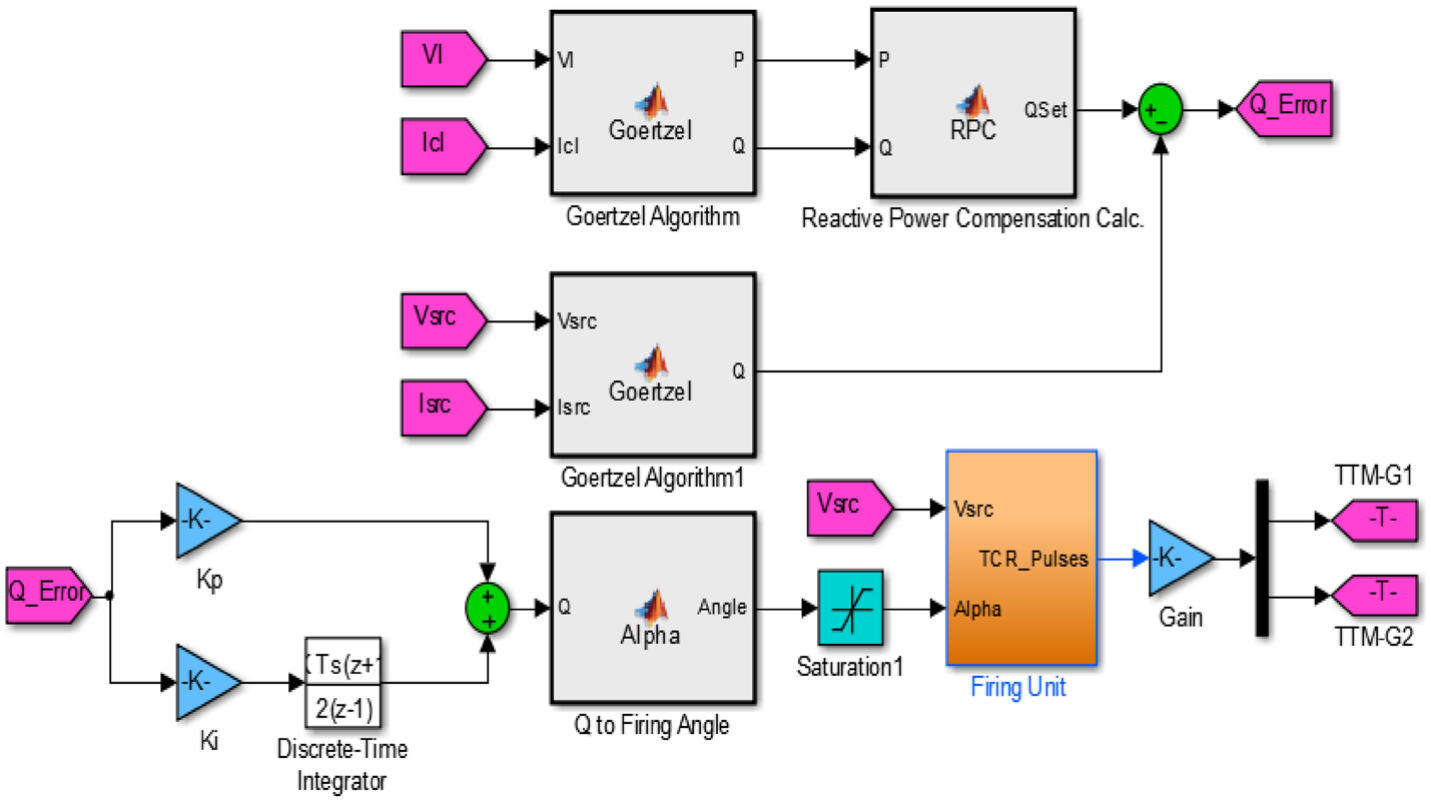
The structure of measurement and controller blocks of FC–TCR

The structures of control and measurement blocks are given in Fig. 3. The setting values for three different load groups and results obtained from the simulation for each operating states are shown in Table 1. Voltage, current, active, reactive, apparent power, power factor and processing time values obtained both working conditions are given in the same table separately.

According to the Table 2, it can be clearly seen that the SVC system can successfully improve the parameters with respect to the condition of before compensation in the event of using Goertzel and FFT algorithms. Although the parameters of the current, reactive and apparent power and power factor are the same for both algorithms before and after compensations, computation time measured for Goertzel algorithm is significantly less than FFT.

**Table 2:** Simulation results

	Goertzel algorithm	FFT algorithm
*Loads*: 300 W + 200 VAr		
Before compensation		
V	207.000	207.000
A	1.741	1.741
VA	360.555	360.555
W	300.000	300.000
VAr	200.000	200.000
P.F.	0.832	0.832
After compensation		
V	207.000	207.000
A	1.449275	1.449275
VA	300.000	300.000
W	300.000	300.000
VAr	0.000	0.000
P.F.	1.000	1.000
Computation time	0.055	0.080

It can be safely said that Goertzel algorithm is faster and superior than FFT compared with computation times. It is seen in Table 2, as an example computation time is 0.055 s. for the Goertzel algorithm while FFT is 0.080 s. According to the Table 2, while the power factor value of the load is 0.832 lagging before compensation, it is brought just 1.00 value by using Goertzel and FFT algorithms after compensation in case of first load condition. Thus the power factor (cos *φ*) is nearly close to 1.00 and SVC system has led to the improvement of power factor. In order to provide approximate values obtained from both simulation and experimental studies, the selected parameters for the simulations presented in Table 2 are based on as possible as the same values used in the experimental working.

**Fig. 4:**
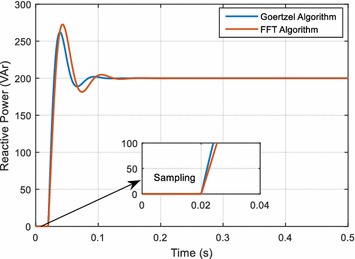
Reactive power response of PI controller

This simulation works are performed using discrete-PI controller. The proportional and integrator gain of the controller is selected as follows: *K*_*P*_: 0.3, *K*_*I*_: 36. Figure 4 shows the reactive power response of PI-SVC with both FFT and Goertzel algorithms. As it seen in Fig. 4, the reference reactive power is 200 VAr. The PI controller with FFT response reaches to reference power after 140 ms with overshoot and the PI controller with Goertzel response reaches to steady state after nearly 110 ms with overshoot.

In this study, only harmonic is analyzed by using Goertzel and FFT algorithms in order to obtain fundamental components of voltage and current to calculate power components and power factor. Any harmonic filter design is not implemented to eliminate any harmonic order.

## Experimental results

**Fig. 5:**
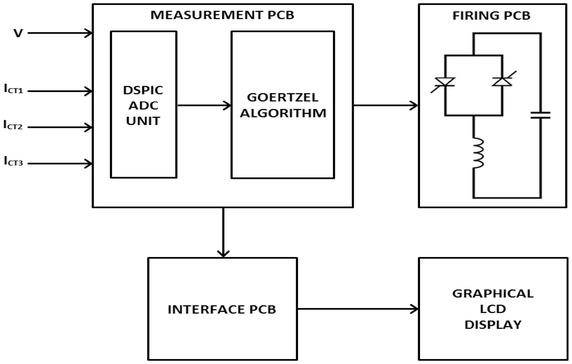
Block diagram of SVC system

In this experimental work, the effect of the new measuring algorithm on the FC–TCR based SVC system is examined for reactive power compensation. The experimental load comprises a coil and resistance connected in parallel. According to the descriptions mentioned above of the SVC system, the general block diagram including voltage and the current measuring unit is depicted in Fig. 5. DsPIC controller is used for measuring the fundamental component of voltage and current by using Goertzel and FFT algorithm in order to calculate active, reactive, apparent power and power factor. Block diagram consist of four parts that are measurement, interface, LCD and firing PCB. Goertzel and FFT algorithms are loaded dsPIC card on the measurement PCB.

**Fig. 6:**
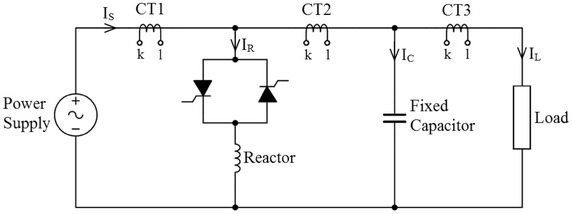
Current transformers and connection point of the circuit

Voltage and current samples are taken at various point of the circuit. Voltage is directly measured, but current is measured via a current transformer. Current transformers and connection points are illustrated in Fig. 6. CT1, CT2 and CT3 are indicated current transformer in the circuit. Voltage and current measured from the circuit are utilized in order to calculate power components and power factor in the load and power sources.

**Fig. 7:**
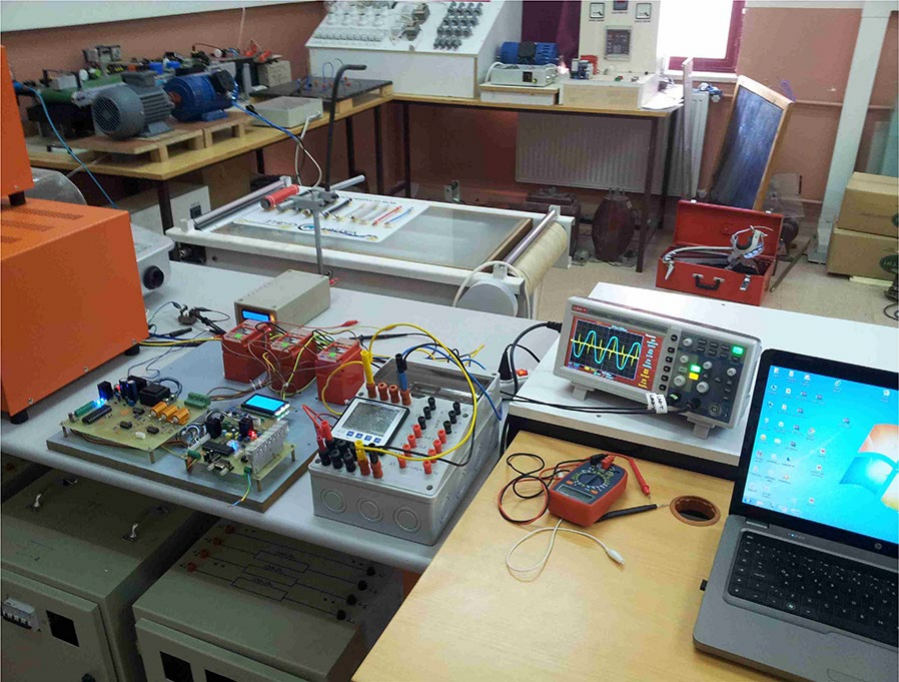
Photograph of the experimental works

**Fig. 8:**
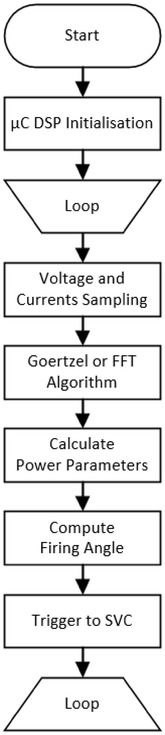
Flow chart of SVC control system

Picture of the experimental study is given in Fig. 7. The dsPIC PCB, firing PCB, LCD, current transformer, different loads, and oscilloscope are shown in the picture. Experimental work is performed with and without SVC system as well as simulation study. PI controller is applied to SVC system. The controlling TCR is the control of firing angle of the thyristor to adjust the current in the reactor. Thyristor firing angles are adjusted so that the current on CT2 is zero. Detailed explanation of how works SVC control system is given as flow chart shown in Fig. 8.

Load values, SVC settings and results obtained for each experimental works are given in Table 3. For the purpose of revealing the performance of SVC system, experimental works are also realized separately for two different conditions. SVC system is considered to be switched on and off respectively. According to the before and after compensation, fundamental components of voltage and currents are measured and calculated by using both Goertzel and FFT algorithms separately. Goertzel and FFT algorithms are programmed and loaded inside the microcontroller unit sequentially. Fundamental components of the power are calculated by using both Goertzel and FFT algorithms respectively.

**Table 3:** Experimental results

	*Goertzel algorithm*	*FFT algorithm*
*Loads*: 300 W + 200 VAr		
Before compensation		
V	207.81	207.20
A	1.680	1.690
VA	349.12	350.18
W	288.85	288.02
VAr	197.41	196.84
P.F.	0.82	0.82
After compensation		
V	207.81	207.20
A	1.390	1.400
VA	288.85	290.09
W	286.77	285.94
VAr	33.24	35.22
P.F.	0.99	0.99
Computation time	0.06	0.085

The setting values and results obtained from the experimental study for each working conditions are shown in Table 3. Voltage, current, active, reactive, apparent power and power factor values obtained both working conditions are given in the same table separately. 300 W resistive and 200 VAr inductive load is used in the experiment. According to the Table 3, it can be clearly seen that the SVC system can successfully improve the parameters in accordance with the condition of before compensation in the case of using Goertzel and FFT algorithms. As can be seen from the Table 3, current, reactive, apparent power and power factor values obtained from both Goertzel and FFT algorithms are almost very close to each other for three experiments in the case of before and after compensation. While the power factor value of the load is 0.82 lagging before compensation, it is brought just 0.99 values by using Goertzel and FFT algorithms after compensation in case of firs load condition. Similar results are obtained in the other load groups. At the same time, current, apparent and reactive power values are reduced to desired values by using both algorithms in case of after compensation. It is clearly said that power factor of the load is successfully improved by the use of SVC system.

In addition to that, voltage, current, active, reactive and apparent power and power factor values are displayed on the screen by the use both algorithms before and after compensation respectively.

**Fig. 9:**
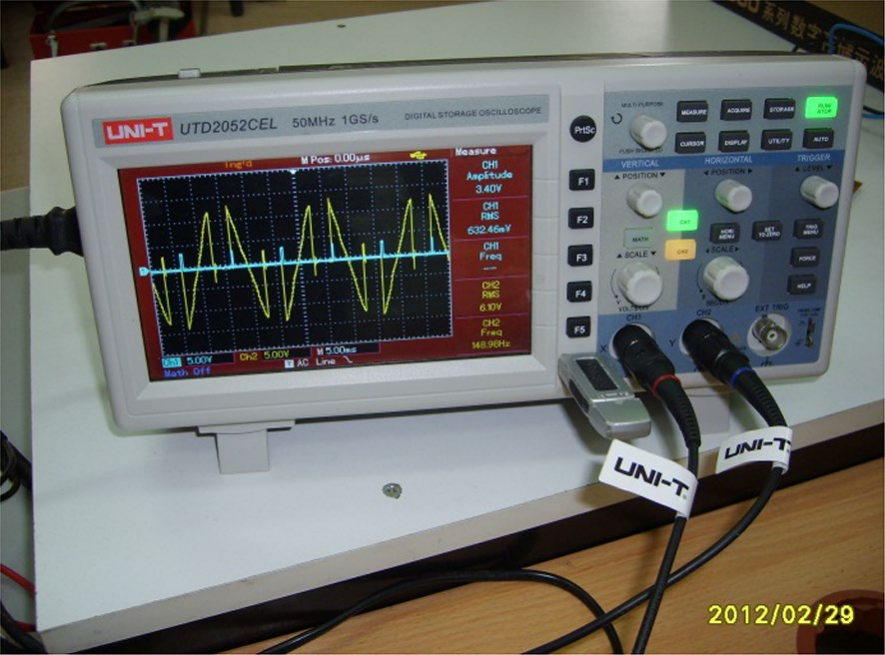
The waveforms of thyristor firing pulse and reactor voltage

The waveforms of thyristor firing pulse and reactor voltage captured by oscilloscope screen are shown in Fig. 9. Firing angles of the thyristors are adjusted according to the calculation of reactive power by using the current measured at the point of CT2. Thyristor firing pulses are indicated with blue colors while yellow colored signals are displayed for the waveform of voltage on the reactor.

**Fig. 10:**
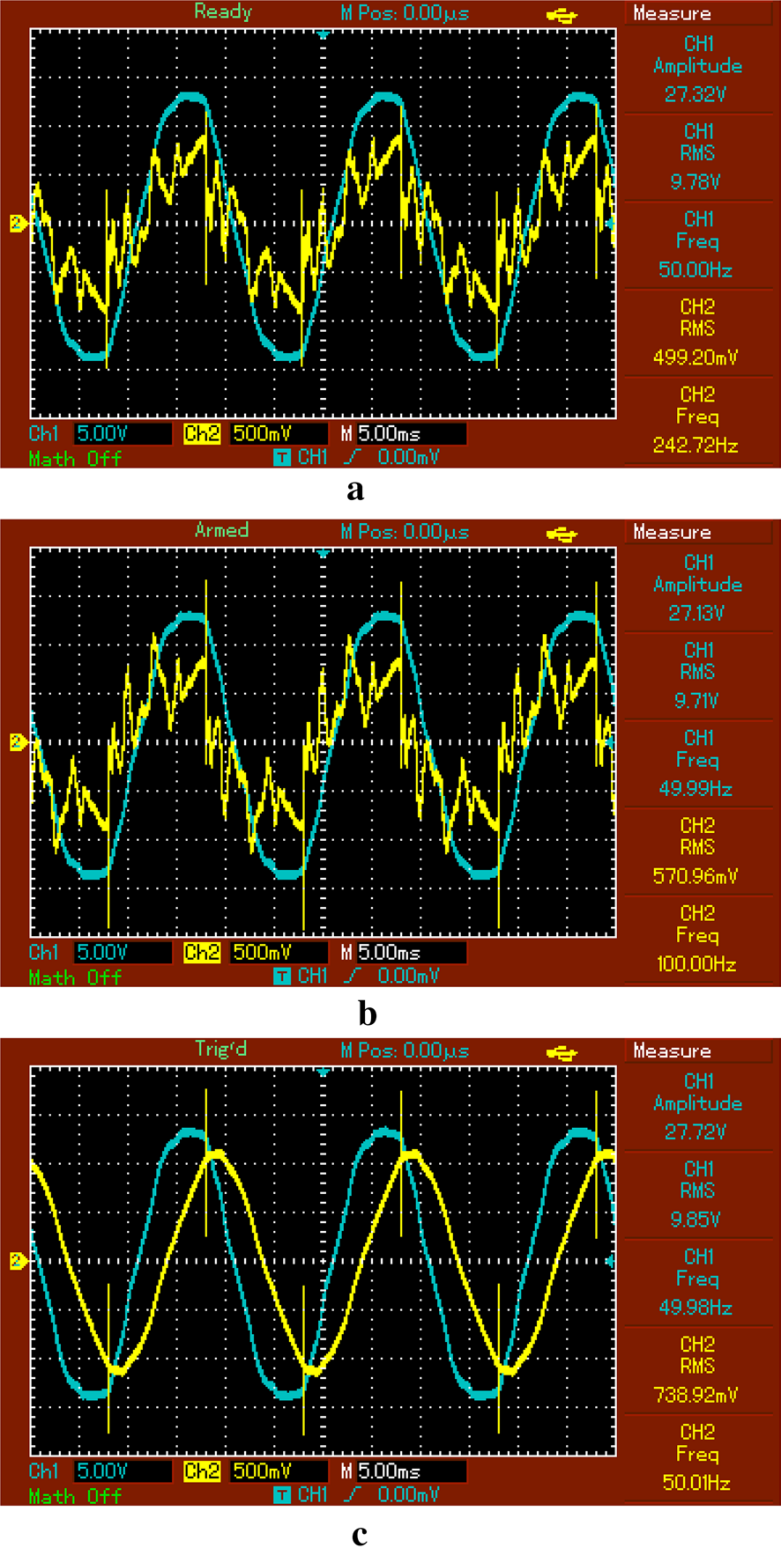
Waveforms of current and voltage signals at the power supply (**a**), CT2 (**b**) and load (**c**)

The waveform of the voltage and current at CT1, CT2 and CT3 points are captured by the oscilloscope and shown in Fig. 10a–c indicate the waveforms of voltage and current measured at the point of power supply, after thyristor and load respectively. While blue colored signal shows the waveform of the voltage, the current waveform is indicated with yellow signals. Both signals contain harmonics. Active, reactive, apparent power and power factor values are calculated according to the signals from each point. However, power components and power factor values in the case of with SVC are calculated with respect to the fundamental parameters of voltage and current analysed by using Goertzel algorithm. Harmonic filter for any harmonic order is not designed in the experimental study.

## Conclusions

In this experimental work, a new method is developed for the FC–TCR based SVC system. In this method, Fundamental components of voltage and current are obtained from the harmonic content network by using Goertzel algorithm instead of FFT. These values are utilized for the calculation of active, reactive, apparent power and power factor for the use of SVC system in order to achieve fast and accurate compensation. This study is realized both simulation and experimental. Simulation studies have shown that the computation time measured by using Goertzel algorithm is faster than FFT algorithm. In this way, power components and power factor values are measured and calculated by minimal hardware and reduced computational time through the use of Goertzel algorithm instead of classical FFT. Power measurement and calculation by utilizing Goertzel algorithm is applied to the FC–TCR based SVC system for the first time in this experimental study.
